# Effectiveness of a web-based health risk assessment with individually-tailored feedback on lifestyle behaviour: study protocol

**DOI:** 10.1186/1471-2458-12-200

**Published:** 2012-03-19

**Authors:** Eva K Laan, Roderik A Kraaijenhagen, Niels Peek, Wim B Busschers, Marije Deutekom, Patrick M Bossuyt, Karien Stronks, Marie-Louise Essink-Bot

**Affiliations:** 1 Department of Public Health, Academic Medical Center, Amsterdam, The Netherlands; 2 NDDO Institute for Prevention and Early Diagnostics (NIPED), Amsterdam, The Netherlands; 3 Department of Medical Informatics, Academic Medical Center, Amsterdam, The Netherlands; 4 Department of Clinical Epidemiology, Biostatistics and Bioinformatics (KEBB), Academic Medical Center, Amsterdam, The Netherlands

**Keywords:** Ehealth, Lifestyle behaviour, Health risk assessment, Worksite health promotion

## Abstract

**Background:**

Physical inactivity, unhealthy dietary habits, smoking and high alcohol consumption are recognized risk factors for cardiovascular disease and cancer. Web-based health risk assessments with tailored feedback seem promising in promoting a healthy lifestyle. This study evaluates the effectiveness of a web-based health risk assessment with individually-tailored feedback on lifestyle behaviour, conducted in a worksite setting.

**Methods/Design:**

The web-based health risk assessment starts with a questionnaire covering socio-demographic variables, family and personal medical history, lifestyle behaviour and psychological variables. Prognostic models are used to estimate individual cardiovascular risks. In case of high risk further biometric and laboratory evaluation is advised. All participants receive individually-tailored feedback on their responses to the health risk assessment questionnaire. The study uses a quasi-experimental design with a waiting list control group. Data are collected at baseline (T0) and after six months (T1). Within each company, clusters of employees are allocated to either the intervention or the control group. Primary outcome is lifestyle behaviour, expressed as the sum of five indicators namely physical activity, nutrition, smoking behaviour, alcohol consumption, and symptoms of burnout. Multilevel regression analysis will be used to answer the main research question and to correct for clustering effects. Baseline differences between the intervention and control group in the distribution of characteristics with a potential effect on lifestyle change will be taken into account in further analyses using propensity scores.

**Discussion:**

This study will increase insight into the effectiveness of health risk assessments with tailored feedback and into conditions that may modify the effectiveness. This information can be used to design effective interventions for lifestyle behaviour change among employees.

**Trial registration:**

Dutch Trial Register NTR8148.

## Background

Most people in western European populations do not meet current recommendations for healthy behaviour, in terms of sufficient physical activity, healthy dietary habits, limited alcohol consumption and non-smoking [1]. For example, in 2008, 37% of European adults aged ≥ 15 years was insufficiently active, i.e. less then 150 min of moderate physical activity per week and the prevalence of daily tobacco smoking among this population was nearly 29%.

In the Netherlands, in 2007, according to the Dutch Healthy Physical Activity Guideline (30 min/day, 5 days/week at moderate intensity level) 41% of the population aged ≥ 18 years was insufficiently active, and 84% was insufficiently active according to the cardio-respiratory fitness guideline of 20 min/day, 3 days/week at high intensity level [2,3]. Results of the Dutch national food consumption survey in 2003 showed that, among young adults (19-30 years), only 5.5% of men and 0.2% of women consumed the recommended amount of vegetables (150 g/day) and only 7-8% consumed the recommended amount of fruit (200 g/day) [4]. Smoking and drinking are also still common behaviours. In 2008, 27% of the population aged ≥ 15 years was smoking, and 14% of men and 11% of women aged ≥ 12 years were drinking too much alcohol [5].

Unhealthy behaviour is known to be a risk factor for chronic conditions such as diabetes, cardiovascular diseases and cancer [5]. Therefore, promoting healthy behaviour has become a central goal of public health policy. Various programs have been developed to promote healthy behaviour and lifestyle change. Offering health promotion interventions via the worksite allows reaching large groups of adults, because most spend a large portion of their waking hours at their workplace [6]. Health promotion via the worksite has the additional advantages of enabling the introduction of social support, and making use of a natural social network for peer support [7]. Health promotion via the worksite may also offer opportunities to make environmental and structural changes that support healthy behaviour [8]. A healthier lifestyle, with the prevention of related (chronic) diseases, not only benefits employees and society, but also employers, in the form of less sickness absence and lower productivity losses [7].

The health risk assessment (HRA) is a commonly used instrument in the promotion of healthy behaviour [9]. A recent review showed that a HRA is useful as a gateway intervention to broader worksite health promotion programs [9]. HRAs screen for risk factors of disease and provide feedback on those risks. Many HRAs now make use of the Internet, which allows delivering individually-tailored interventions [10]. For example, within the worksite setting, emails and web pages can reach broad and diverse employee populations [11]. With web-based HRAs, employees are also able to apply the program in privacy, e.g. at home, and at a time that suits them [12]. However, although HRAs are promising, there is limited knowledge about what actually makes them work [8].

The primary aim of this study is to evaluate the effectiveness of a web-based HRA with individually-tailored feedback conducted in a worksite setting, on lifestyle behaviour. A secondary aim is to explore conditions that could modify the effectiveness in specific settings or subgroups. The results of this study will contribute to the evidence base for web-based HRAs with tailored feedback aimed at changing health behaviour.

## Methods/Design

### Study design

A quasi-experimental study will be conducted with a waiting list control group, carried out in a worksite setting. The study population consists of employees of companies that embedded the HRA in their corporate health management strategy. Data are collected at baseline (T0) and after six months (T1). Data collection started in April 2011. According to Dutch law, medical ethical approval of the study was not required, as confirmed by the Medical Ethics Committee of the Academic Medical Centre of the University of Amsterdam. All necessary precautions to protect privacy of subjects were taken.

### Intervention

The intervention is called the 'Prevention Compass'. It has been developed by the NDDO Institute for Prevention and Early Diagnostics (NIPED) in Amsterdam. The Prevention Compass consists of a web-based HRA with tailored feedback. Participation in the intervention is on a voluntary basis. All current employees of a participating company are invited to participate in the intervention. Excluded are pregnant women or women with a pregnancy in the past 6 months because, in this group, such measurements could result in abnormal values.

#### Invitation for the intervention

Employees are invited through a letter sent to their home address. An information leaflet with a description of the intervention is enclosed. The invitation letter informs employees that participation is voluntary, involves no personal costs, and that all personal data are treated confidentially, meaning that no data are shared with their employer, and that the data are used in an anonymous form for the present study only. No written informed consent is obtained, due to the fact that the intervention is online. Completion of the questionnaire is regarded as informed consent to participation. The invitation letter contains an activation code for registration at the intervention's website http://www.preventiekompas.nl. For each participant, a personal online health portal is made available. Here participants can fill in the questionnaire, order a box with measurement tools (see below), and view their results of the HRA. After registration, participants receive a personal number for secure access to their portal. In case of no response to the letter of invitation, invitees receive a single reminder after three weeks, in the form of a second letter to their home address.

#### Web-based health risk assessment

The web-based HRA starts with a questionnaire, covering socio-demographic variables, family and personal medical history, lifestyle behaviour (physical activity, smoking behaviour, alcohol consumption, dietary pattern) and psychological variables (stress, burnout, depression). Individual scores are used to estimate cardiovascular disease risk using validated risk scores. All risk calculations are based on prevailing practice guidelines, including the European and Dutch guidelines for cardiovascular risk management [13,14]. Individuals at increased risk for cardiovascular disease, diabetes and kidney disease are advised to perform additional biometric and laboratory evaluation. For this, participants can order a box with measurement tools via their portal. This box includes a sphygmometer, a measuring tape and instruction cards for measurement of blood pressure and waist circumference. Participants also receive a lab box with materials to collect a urine sample for cardio-metabolic measurements. All participants who ordered the lab box visit a central facility where urine samples can be handed in, and a blood sample is taken for analysis of total cholesterol, LDL-cholesterol, HDL-cholesterol, triglycerides, glucose, creatinin, urinary albumin to creatinin ratio, and HbA1C. Results are electronically transferred to the central HRA database. After additional measurements, results are used to recalculate the cardio-metabolic risk, define kidney function using the MRDR formula, i.e. to estimate the glomerular filtration rate (eGFR) and albumin-creatinin ratio and detect (pre)diabetes based on glucose and HbA1C values.

For system security and data protection reasons, personal identification data and HRA data are stored on separate servers. An electronic firewall is placed between the servers and the Internet. Only participants certified by ID and password are able to access the servers.

### Tailored feedback

#### Theoretical framework for lifestyle change

The tailored feedback provided as part of the intervention is based on Prochaska's transtheoretical model [15]. According to this model, behavioural change is a process consisting of five subsequent phases: precontemplation (no intention of behaviour change between one and six months), contemplation (considering behaviour change between one and six months), preparation (getting ready to change behaviour in the next month), action (performing the change in behaviour), and maintenance (steady stage of behaviour change reached). In the feedback, individuals receive a health plan that fits with their current stage of change. Participants in earlier stages receive information to motivate them to change their lifestyle and participants in later stages receive concrete action plans how to change their lifestyle behaviour.

#### Tailored feedback

Participants receive tailored feedback based on their responses to the HRA questionnaire and additional measurements if indicated. The feedback contains a health risk profile divided into four domains: lifestyle, psychological, social (including work-related aspects) and physical, and each of these divided into sub-domains (Table 1). For each of these domains the assessed health risk is explained using a simple grid labelled by one of three colours: green representing 'normal risk', orange representing 'moderately elevated risk' and red representing 'seriously elevated risk'. In addition, threats associated with elevated risk (orange and red categories) and potential gains of taking preventive action are explained. The feedback concludes with an overview of actions the participant can take. The number of possible actions depends on the outcome of the HRA. All options include suggestions from trusted external parties, where participants could go for support for the action they want to take. The suggestions are made to fit with their expressed preferences, such as for guided vs. non-guided interventions.

**Table 1:** Health-related domains and sub-domains on which participants of the health risk assessment receive tailored feedback

Lifestyle domain	Psychological domain	Social domain	Physical domain
Physical activity	Stress	Workability/productivity	Blood-pressure*

Smoking behaviour	Burn-out	Work satisfaction	Lipids*

Alcohol consumption	Depression	Work-home balance	Blood sugar*

Nutrition	Anxiety	Interaction with colleagues	Kidney function*

			Body weight and fat distribution

			Complaints of arm, neck and shoulder

*Only if additional biometric and laboratory evaluation are performed.

In summary, differentiations are made between the stage of motivation (see 'Theoretical framework for lifestyle change'), the preferences for professional guidance (yes/no), actions in groups or alone, actions outside or close to home, and actions via internet, telephone or face-to-face. In case of high risk (red category), the feedback includes referral for further medical evaluation and treatment if necessary.

### Evaluation study

#### Study population and procedure

All employees aged ≥ 18 years from companies participating in the study are eligible for participation in the evaluation study. Individual participation in the study is on a voluntary basis. Employees in the intervention group receive an invitation for participation in the HRA at baseline. Their completion of the lifestyle items in the questionnaire of the HRA serves two purposes: it belongs to their HRA but also acts as a baseline measurement of health behaviour for the evaluation study. Six months later participants receive an invitation for a short, electronic lifestyle questionnaire that serves as follow-up measurement.

**Figure 1:**
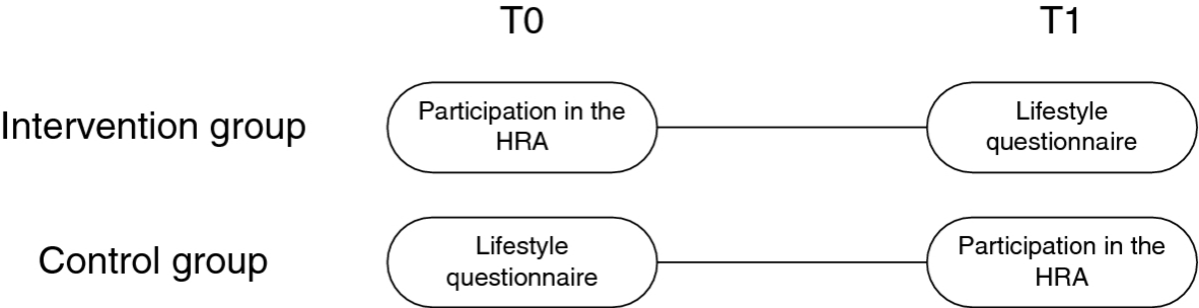
**Schematic overview of the participation**.

Employees in the control group receive an invitation for the short, electronic lifestyle questionnaire at baseline. In this group this questionnaire serves as the baseline measurement. Six months later control group participants receive an invitation to take part in the HRA. The completion of the lifestyle items in the questionnaire of the HRA now serves as follow-up measurement (Figure 1).

##### Allocation to the intervention or control group

Individual allocation may lead to contamination between the groups in the case that collaborating employees are allocated to different groups; this could then imply that the results are influenced by factors other than the intervention. Therefore, we rely on allocation by cluster. Within each company, clusters of employees with comparable activities and of comparable size are allocated to either the intervention or the control group.

Due to practical limitations, random allocation of clusters to the intervention and control group is not possible. Both the intervention and the study have to be embedded in the corporate health management strategy of the participating companies. Allocation of units to the intervention and control group will follow the roll-out procedure of the company, i.e. by geographical region and mainly based on practical considerations of the company management like current reorganization. Although the allocation procedure is not random, confounding is unlikely; conversations with the management teams of companies confirm that clusters could not obtain priority in the allocation procedure.

Furthermore, effects of this limitation on the estimate of the effect of the intervention will be minimized by adjusting for baseline characteristics of clusters in the statistical analysis (see 'Statistical analysis').

##### Invitation for the lifestyle questionnaire (control group)

Employees are invited through a letter to their home address to complete the lifestyle questionnaire via Internet. An information leaflet about the evaluation study is enclosed. The invitation letter informs employees that participation is voluntary, that all personal data are treated confidentially, and that no result is shared with their employer and only used in anonymous form for the present study. The invitation letter contains a unique number for access to the questionnaire via the website. In case of no response to the letter of invitation, participants receive a single reminder after three weeks, in the form of a letter to their home address.

#### Measurements

The responses to the HRA questionnaire are used for two purposes. First, the responses form the basis for generation of the tailored feedback. Second, the responses to the lifestyle items are used to analyse the outcome measures of this evaluation study.

Primary outcome is lifestyle behaviour, expressed as the sum of five indicators (physical activity, nutrition, smoking behaviour, alcohol consumption and symptoms of burnout). For each indicator, adherence to the Dutch guidelines (yes/no) is determined. The magnitude of change in lifestyle behaviour measured after six months determines whether or not the intervention is successful.

##### Physical activity

Physical activity is measured with two items derived from the Dutch version of the International Physical Activity Questionnaire (IPAQ) [16]. These two closed-ended items provide information on the frequency (days per week) and duration (minutes) spent on moderate intensity and vigorous physical activity in a normal week.

The public health recommendation for physical activity in the Netherlands, the Dutch Healthy Physical Activity Guideline (*Nederlandse Norm Gezond Bewegen*), recommends physical activity for 30 min (or more) per day at a moderate or vigorous intensity level, for at least 5 days a week [3]. Adherence to this guideline is measured with one additional item about the number of days that the participant is physically active for 30 min (or more) per day at a moderate or vigorous intensity level.

To generate the feedback, the total duration of physical activity in min/week is calculated, collating moderate and vigorous activities. Additionally, total physical activity duration is dichotomized into ≤ 150 min/week or ≥ 150 min/week, as a second way of reflecting adherence to the Dutch Healthy Physical Activity Guideline.

For the evaluation study, adherence to the cardio-respiratory fitness guideline (Fitnorm) is also analyzed. This guideline recommends physical activity for 20 min (or more)/day at a vigorous intensity level, for at least 3 days/week [2]. Adherence to this norm is analyzed using the questions described above. Total physical activity duration is dichotomized into ≤ 60 min/week or ≥ 60 min/week. The cardio-respiratory fitness guideline is not used in the tailored feedback of the intervention.

##### Nutrition

Different aspects of the dietary pattern are taken into account, as defined by the Dutch Dieticians Cooperation: fruit and vegetables, saturated fat intake, intake of sugar, and fish.

Items concerning fruit and vegetables are based on a standard nutrition questionnaire of the Dutch Municipal Health Service [17]. Participants are asked to report their fruit intake in pieces of fruit per day in a normal week. They also are asked to report the number of serving spoons of vegetables per day in a normal week. In the Netherlands, a daily intake of at least 2 pieces of fruit and 200 g of vegetables (4 spoons per day, defining a serving spoon of vegetables as 50 g) is the public health recommendation [18].

Saturated fat intake is measured with items developed in expert meetings. Participants are asked to report the amount of snacks, use of butter, cheese and meat (portions/day), dairy products and sweets. To generate the feedback each answer has a score that cumulatively leads to a total score for saturated fat intake. Experts defined a cut-off value that is used to determine the colour (green/orange) used in the feedback.

The intake of sugar is based on the amount of teaspoons of sugar, as well as sweet drinks and sweet snacks consumed per day. Again, to generate the feedback, each answer has a score that cumulatively leads to a total score for intake of sugar; a daily intake of 40 g or less is recommended.

Also noted are the frequency of breakfast (days/week) and the frequency of fish (portions/week). The public health recommendation of frequency of breakfast is every day, thus 7 days a week [18]. For fish, this recommendation includes fish consumption at least two times a week, of which at least one portion fatty fish [18]. All aspects of the dietary pattern are dichotomized according to the guidelines, to generate the feedback.

##### Smoking behaviour

Smoking behaviour is measured by items from a questionnaire of the Dutch Expert Centre on Tobacco Control (STIVORO) [19]. To generate the feedback, participants are categorized into non-smokers, ex-smokers, incidental smokers, daily smokers and heavy smokers. A heavy smoker is defined as smoking 10 or more units of tobacco per day. The evaluation study only incorporates the questions if the participant is currently smoking (yes/no), and then the kind of tobacco, and the units of tobacco per day.

##### Alcohol consumption

Alcohol consumption is measured in units of alcohol per day based on a standard alcohol questionnaire of the Dutch Municipal Health Service [20]. For men and woman different limits for excessive alcohol consumption are used; for men alcohol consumption up to 21 units/week and for women alcohol consumption up to 14 units/week is recommended as maximum. Furthermore, alcohol dependence is measured using the 'five-shot' questionnaire [21]. This questionnaire gives an initial indication concerning alcohol dependence via 5 questions. Each answer has a score that contributes to a total score of alcohol dependence. The total score has a defined cut-off value for alcohol dependence. To generate the feedback, the units of alcohol per day and the score of the five-shot questionnaire are dichotomized according to the norm.

##### Burnout

The UBOS questionnaire is used for symptoms of burnout [22]; this questionnaire is the Dutch version of the the Maslach Burnout Inventory [23]. Each answer has a score that contributes to a total score of indication for burnout. The total score has a defined cut-off value. To generate the feedback, indication for burnout is dichotomized according to this value.

#### Determinants of behavioural change

Additional determinants of health behavioural change are measured, as described below.

##### Intention to behaviour change

The intention to change behaviour is measured per lifestyle variable. Answer options are based on the different phases of Prochaska's 'Stage of Change' model [15], and are formulated as: 'I don't have any intention to change my lifestyle between one and six months', 'I am considering to change my lifestyle within between one and six months', 'I'm getting ready to change my lifestyle in the next months', 'I'm already working to improve my lifestyle', and 'I have reached the intended lifestyle behaviour and still perform it'.

##### Self-efficacy

Self-efficacy is measured by one newly developed item where participants have to indicate to what extent they feel able or unable to change their lifestyle behaviour if necessary. This item is formulated as: 'I am able to change my lifestyle behaviour if necessary'. Answers are rated on a 5-point Likert scale ranging from 1 (totally agree) to 5 (totally disagree).

##### Social support

Social support is measured by three items; two reflecting the support for participating in a HRA with tailored feedback from the respondent's work environment (colleagues and managers), and one reflecting the support from the respondent's partner and/or family. Respondents are asked to complete the statements: 'According to my colleagues, participation in a HRA with tailored feedback is useful', 'According to my manager, participation in a HRA with tailored feedback is useful', and 'According to my partner and/or family, participation in a HRA with tailored feedback is useful'. Answers are rated on a 5-point Likert scale ranging from 1 (very useful) to 5 (totally useless) and an additional answer option 'I don't know'.

#### Confounding variables

Non-random allocation to the intervention and control group may result in an unequal distribution of the characteristics of employees and/or companies and their units, which can influence the changes in lifestyle behaviour. Measured potential confounders of the change in lifestyle behaviour include age, gender and educational level, years of work at the current company, years in current function, and part-time or fulltime employment.

#### Statistical analysis

Descriptive statistics will be used to describe and compare baseline characteristics of participants and clusters in the intervention and control group. Any baseline differences with a potential effect on lifestyle change will be taken into account in further analyses using propensity score weighting. To estimate the effect of the intervention on lifestyle behavioural change logistic regression analysis (for binary outcome measures) and linear regression analysis (for continuous outcome measures) will be used. To correct for a potential clustering effect, multilevel analyses will be performed. In case the follow-up measurement result in a considerable amount of missing data, multiple imputation methods will be used.

#### Power analysis

The sample size calculation is based on the primary outcome, lifestyle behaviour, expressed as the sum of five indicators. We consider a difference in a range of 0.3 to 0.5 points more behavioural change in the intervention group compared to the control group as a meaningful effect (power of 80%; one-sided significance level, 0.05). With an intra-cluster correlation of 0.05 (as in a previous study among employees [24]), and an average of 30 employees per cluster (based on 100 eligible employees per cluster, 30% initial participation), we must include at least 17 clusters per group, thus a total of 1020 employees, to find a difference of 0.3 points. To find a difference of 0.5 point we have to include at least 7 clusters per group, thus a total of 420 employees.

## Discussion

The study presented here is designed to evaluate the effectiveness of a health risk assessment with tailored feedback conducted in a worksite setting, on lifestyle behaviour. The rationale for this study is the wide availability and use of worksite HRAs, in combination with the lack of evidence concerning their impact on health behaviour.

Results may be relevant for future development and implementation of web-based interventions aimed at improving lifestyle behaviour relevant for prevention of cardiovascular diseases and cancer. In the present study, the broad approach of the evaluated HRA, and the fact that feedback is tailored to participants' characteristics, make it of potential interest for all individuals and not only for those at high risk. It creates awareness of unhealthy behaviour before chronic disease symptoms are present, by providing information about (un)healthy behaviour in the health recommendations. This is a crucial step for those not yet ready for behavioural change.

The fact that the present evaluation is performed in a real-life setting is a strength, because this provides a realistic estimate of effectiveness. The controlled design allows for evaluation of the effect, in addition to autonomic change in lifestyle behaviour.

Potential sources of bias include the Hawthorne effect, registration bias, and carry-over bias. The Hawthorne effect can occur in individuals who change their behaviour due to the attention they receive from, e.g. the researchers. In this case, however, the effect is probably limited because the intervention is mostly provided online with little contact with the researchers. Bias due to participants giving socially desirable answers is a possibility when using self-report questionnaires; although we aim to minimise such bias by formulating the items in a neutral way and using validated questionnaires where possible, it cannot be excluded. Finally, we aim to limit carry-over bias by the use of cluster randomization.

Taking into account the benefits of a worksite setting and the web-based delivery of the HRA, we believe this HRA is applicable to and attractive for the population at large. This study has the potential to make a substantial contribution to the development of effective interventions for lifestyle behaviour change among a working population. Results are expected to be available in 2013.

## Competing interests

Roderik A Kraaijenhagen is medical director and co-owner of NIPED. This institute developed and currently markets the studied program in the Netherlands. NIPED assists several research programs for scientific quality assurance purposes that are conducted by academic institutions. The present study is an example of such a project. During this research, Eva K Laan was employed at NIPED on a part-time basis (4 h/week) for which she received a salary.

## Authors' contributions

MD designed and wrote the original proposal, supported by KS, PB and RK. EL further developed the study protocol and is responsible for data collection, data analysis, and drafting of the final research report, supervised by MLE, NP and RK. All authors participated in discussing the design of the study and developing the research protocol. All authors have read and approved the final manuscript.

## Pre-publication history

The pre-publication history for this paper can be accessed here:

http://www.biomedcentral.com/1471-2458/12/200/prepub
